# On the History and Properties, Chemical and Medical, of Tobacco

**Published:** 1840

**Authors:** 


### ON THE HISTORY AND PROPERTIES, CHEMICAL AND MEDICAL, OF TOBACCO, a Probationary Essay presented to the Faculty of Physicians and Surgeons, Glasgow. By *Henry Wilson Cleland*, M.D., Lecturer on Medical Jurisprudence in the School of Medicine, Portland Street. (A Candidate for Admission into that Body.) Quarto, pp. 68. Glasgow, July 1840.

THIS is a book of the right sort. One like Burton's Anatomy of Melancholy, or Henderson on Wines, or, si liceat magnis componere parva, John Knox's (*not* the reformer's) Curator's Vade Mecum. Dr. Cleland has taken up smoke in a right substantial fashion, and if so light a matter could be made so much of, what could he not do with a weightier! We would recommend the faculty of physicians and surgeons of Glasgow to elect him forthwith. We would bet a pound that the faculties of the whole faculty would be gravelled to produce such another work.

Dr. Cleland apologises for choosing tobacco for the subject of his thesis. Upon our honour we acquit him. His essay is worth all the theses we have ever seen. What can a young man know of tetanus, or fever? He can, at the best, but make a dull compilation, and if there is originality it is probably pertness. But tobacco and a pipe may be *puffed* by the inceptor candidate as well as by the "sad and learned" doctor of threescore, nay, perhaps, a great deal better by young lungs than by old ones. Then make no apology, Dr. Cleland, we beg.

The Doctor tells us, in his Preface, that he might, if he had liked, have written a book entirely medical and supremely dull. Or, he might have compiled one with not a smack of physic about it. He has done right to do neither.

"We have chosen," quoth he, "to pursue a middle course; and while we hold, as a basis, the medical properties of the plant of which we propose to treat, we will illustrate, as much as possible, details of a purely scientific nature, by those perhaps less instructive, though more brilliant and pleasing passages, which shed such a glow of light over the departments of literature. We are the more inclined to adopt this view, from the splendid and vivid galaxy of literary characters which adorned Great Britain in the latter end of the sixteenth, and the commencement of the seventeenth centuries—the very period at which this drug was introduced, and very much employed in this country. The poetical writers of that time, too, especially those of the dramatic department, were not, as is too commonly supposed, mere imaginative visionaries, who 'piped on reeds or whistled on straws;' but men endowed with strong powers of observation, of perception, and of judgment; and who had the boldness and hardihood to express whatever they thought, on every subject, in a manner quite unexpected, but no less welcome in those ages of comparative tyranny.

Luxury and abuses of every shape were then, as they have always been, the proper objects of satire and reprehension; and these were furnished, as was to be expected, in a direct proportion to the extent into which the former had been indulged, or the length to which the latter had proceeded. Amongst these, none were handled so caustically, or in so complete a manner, as the abuses in medicine; and at no part, it is believed, in the literature of any time, are we so much indebted for insight into medical doctrines and medical practice to the dramatists and satirists, as at that of which we are now speaking; and if it be true what has been said of the great Marlborough, and of the still greater Chatham, that their knowledge of English history was mainly derived from the perusal of Shakspear, it might with perfect safety be averred, that by the careful and rigid study of the works of such men as Jonson, Beaumont, Fletcher, Massenger, and Burton, a mass of medical facts and medical opinions might be obtained, infinitely superior to those contained in the works of the physicians of the times in which they lived, and which, even at this day, might put to shame the *passes* of the mesmerists, or the *infinitesimals* of Hahnemann. Indeed, it is to these, and not to the professed writers on medicine, that we must look for our information regarding, not merely the customs and peculiarities of medical men—a subject perhaps treated in a superior manner subsequently by Moliere—but, in reality, respecting the routine of that and of the preceding century, and more especially the opinions of the ancient physicians; for while Helmont, Borrichius, Anthony, Fludd, and a multitude of others, were revelling in all the absurdities of a universal remedy, and of

————'That stone which here below
Philosophers in vain so long have sought;'

While Riverius and Ferrand were attributing all that they could not with facility comprehend, to the very convenient alternative of 'certaine occult hidden qualities;' and while Foreman, Hoyden, Withers, and an innumerable host beside, were

'Trowling the Trine, the Quartile, and the Sextile,
Platic aspect, and Partile, with his Hyleg,
Or Alchochoden, Cuspes Horoscope;'

Or,

'Intent on the erection of a scheme
For my great madam's monkey, when 't has ta'en
A glyster and bewrayed the Ephemerides.'

Jonson had already created a Subtle and a Sly, a Face, a Sordido, and a Sir Epicure Mammon."

Our readers may already see the man they have to deal with. Hearty in the cause, he lavishes illustrations and quotations most profusely on his text, and we might almost say of him

"Schœnobates, augur, medicus, magus, omnia novit."

We intend accompanying Dr. Cleland through his thesis, and giving our readers a spice of what he offers. We cannot of course quote all, but what we shall give will afford a sample, at all events, of what remains.

### HISTORY OF TOBACCO.

Dr. Cleland may well observe that a *cloud* of obscurity hangs over its origin. Nothing great but has a traditionary source, and there is as much dispute on the spot that may claim tobacco for its own, as on the natal place of the first Romans. Dr. Cleland, however, is a greater than Niebuhr in his way. The Doctor laments the lies that have been told, and repeats the pithy sarcasm of Walpole, that "as the readers of history love certainty, it is pity the writers do not."

Keeping out of view, however, says the Doctor, the celestial origin fancifully assigned to it by Sylvester and Thorius, and the diabolical parentage which was conferred on it, in jest, by Ben Jonson,* and spitefully by the Abbé

* Vide Song at the end of the Masque of "The Gipsies Metamorphosed," works, vol. vii. p. 413; Lord Bacon supposed that, into "the ointment which witches use," tobacco entered as an ingredient. (*Sylva Sylvarum, cent.* 10, 976.) In the

"Proclamation,
Or approbation,
From the king of execration
To every nation
For tobacco's propagation,"

the Water Poet assigned it an infernal descent; and in a proeme to a reprint of Skelton's "Elinour Rummin," the deceased laureate's ghost is made to say,—

"For in King Harry's time,
When I made this rime,
Of Ellinour Rummin,
With her good ale tunning,
That time did not know
To puff and to blow

Nissino, we observe that claims for the distinction have been raised in behalf of the four great continents of the world—of Asia, Africa, Europe, and America.

Chardin, continues our author, who was in Persia about the year 1670, relates in his travels, that tobacco had been cultivated there from time immemorial; and Murray is persuaded, that long before the discovery of the Western World, the smoking of this herb was in common use among the Mongols and Tartars. Bell of Antermony asserts, that " it is reported the Chinese have had the use of Tobacco for many ages;" an opinion in which Pallas completely coincides, from a consideration of their bamboo pipes, and of the singular mode in which they inhale the smoke. While Rumph, who resided at Amboyna towards the latter portion of the seventeenth century, found it universal over the East Indies, even in countries where Spaniards or Portuguese had never been.

In Savary's " Parfait Negociant," it is stated, that the inhabitants of Persia have been acquainted with tobacco for four hundred years; and he suggests the idea, that the custom was received from Egypt, rather than from the East Indies, where it has been only cultivated since the beginning of the seventeenth century; while Professor Lichtenstein, who lived at the Cape of Good Hope for several years at the commencement of this century, is inclined to assign the *south* of Africa as the primary situation where tobacco was employed. " It is remarkable," he observes, " that the custom of smoking and snuffing certain acrid narcotic herbs was in activity among the Beetjuanen race, long before their intercourse with the Europeans. These customs were probably introduced by slaves of African stock into the West Indies, from which it has come to us. So that in the Beetjuanen we have to respect (verehren) the oldest teachers (Lehrer) of this custom, which for a century has been so universal in Europe."

Liebault, in his " *Maison Rustique,*" (published 1582,) asserts, that tobacco exists naturally in Europe; and that before the discovery of America, he had actually procured some in the wood of Ardennes,—a statement similar to that of Libavius, that it grows wild in the Hercynian forest. And Eulia Effendi mentions in his travels, that he found a tobacco-pipe still in good preservation, and retaining the smell of smoke, imbedded in the wall of a Grecian edifice, which had existed since before the birth of Mahomet; and which, in his opinion, incontestibly proved the antiquity of the practice.

But it is refreshing to see how quickly Dr. Cleland dispatches Chardin,

---

In a piece of white clay
As you do at this day,
Sucking and drinking
A filthie weed, stinking,
Was ne'er known before,
*Till the Devil and the More*
In the Indies did meet,
And each other did greet;
With a health, they desire,
Of stink, smoke, and fier,
Good physick of course,
To cure a sick horse."

Murray, Bell of Antermony, Pallas, Rumph, Savary, Professor Lichtenstein, Liebault, and Eulia Effendi. He proves these chroniclers of smoke in a twinkling to be, like Ferdinand Mendez Pinto, liars of the first magnitude, and tilts them over with as much facility as the Lord of Eglintoun would his own dummy.

America, which makes out so good a case for the original possession of the " great pox," seems to have quite as good claims, or better, to the original ownership of the great weed. For, on the third island belonging to that continent at which Columbus touched, namely, Cuba, and at which he arrived on 28th October, 1492, we are told,* that some of his men who were sent to explore the country, brought back the information, that " by the way they saw many people, who always carried a lighted firebrand to light, fire, and perfume themselves with certain herbs, which they carried along with them;" which is the first notice—imperfect though it must be allowed to be—of the practice of smoking, what was at a subsequent period ascertained to be, tobacco.

But, in spite of M. Merat, who asserts that Europe has the honor of having first *snuffed*, that seems to be the oldest fashion. And in *its* source we may perceive the reason of a practice which, to the uninitiated, appears one of sheer and unmitigated beastliness. There is something divine and inspiring in it, and now we understand why it is that many learned and unlearned persons of our day, never form a resolve or venture a remark without first *shooting* a boxful of dust into their noses. Listen.

In the account of Haytian Mythology, drawn up by a friar, named Roman Pane, who accompanied the "great adventurer" in his second voyage, in 1794, it is related, that one Caracola, who existed before the creation of the sea, went into a house begging some bread; " but the master of the house clapped his hand on his nose, and threw on him a guangaio of *Cogioba*, which he had made that day, and is a sort of powder they take sometimes to purge themselves.† This they take through a cane, half a cubit long, one end whereof they put to their nose, and the other end to the powder, and so snuff it up, which purges them very much."‡ They used this powder also very frequently for the solution of all questions regarding their national affairs, such as, whether they should declare for, or desist from war; and also as a mode of divination regarding a season of plenty or of scarcity: their chief being made drunk with the *Cogioba*, snuffed up his nose, by which

* Life, by his son, Don Ferdinand.

† Notwithstanding this decisive proof of the antiquity of snuff-taking, we find several French writers intent on assigning to it a much more recent origin. For example, Merat, in his " Dictionnaire Universelle de Matiere Medicale," declares " la prise " to be " *tout Européen!* " and the writer of the article " Tabac," in the " Dictionnaire des Sciences Naturelles," observes, that " il paroit que l'usage de l'introduire en poudre dans le nez étoit alors inconnu, et qu'il le fut même encore quelque temps aprés son introduction en Europe."

‡ The history of this Caracola is interesting, since it is the first instance related of a transatlantic mother bringing forth four at a birth; and more especially as shewing the antiquity of what is commonly called the Cesarian Section; " for the said woman," says Roman Pane, " dying in labour, they *cut her open*, and *took out* the said sons."

means "the houses appeared to him to turn topsy-turvy, and the men to go upon their heads," and afterwards declaring the oracles which he pretends to have received from his Cemies, (or idols,) during the state of intoxication.

These Haytians made the most of their snuff, and afforded a hint that some modern physicians would seem to have taken.

"The Haytian doctor is obliged to be dieted as the sick man is, *and so look like him*, which is done thus,—He is to purge himself as the sick man does, which is done by snuffing a certain powder called Cohoba up his nose, which makes him drunk, that he knows not what he does, and so *says many extravagant things*, which they affirm is talking with the Cemies, and that they tell them how the sickness came."

So excellent an idea might be worked out by our clever medical Chevaliers d' Industrie. Dr. Dickson, the ci-devant fallacy man of Cheltenham, should look to this. It might form the basis of his new system. What an immensity of good a bushel of "Irish blackguard" would do the doctor. We dare say, however, he has plenty of it.

Perhaps some of our readers may suppose that *chewing* is, at all events, an European refinement. Not a bit of it. We have been forestalled by the Americans. On the 17th of October, 1502, at the bay of Caravaro, on the coast of Paraguay, the inhabitants, on the approach of the Spaniards, came down beating drums, throwing the salt water towards the Christians—*chewing herbs, and spurting it towards them.*

We have seen the Aborigines of America fumigating themselves, snuffing, and last, not least, chewing the glorious weed. To them must be ascribed the merit of first smoking it, and *pipes* appear upon the scene. Nay such was the genius of that tobacco-loving race that they had invented the cigar!

"The first particular account of the mode in which the herb was smoked, is contained in the second edition of the 'La Historia general y natural de las Indias Occidentales,' published at Salamanca in 1535, by Oviedo. This author mentions, that the principal men in Hispaniola have little hollow sticks, of the shape of the letter Y, the two superior extremities of which they insert into their nostrils, and the single inferior one they hold over the burning leaves. To this instrument, he says, the natives give the appellation *Tabaco*, from which the term at present in use for the plant is obviously derived.* The pipe, however, seems in a short time to have become less universal, or even in some degree obsolete, as in the account of Hispaniola, in 1541, by Hyeronimus Benzone, we learn, that the inhabitants, after drying the herb, enclosed several leaves in one of maize, and having applied a light at one end, drew the smoke into their mouth by the other, and so fond did they appear to be of the state induced by this practice, that there were many 'qui adeo avide et furenter eum (scilicet Taba-

---

* "Here, again, we find Merat at fault; he supporting the exploded notion, that the Spaniards designated the herb tobacco, from Tabaco, a town of New Spain, where they first saw it. (Dict. des Sc. Med. art. Tabac.) And Burnet, although assigning the proper derivation, fails in drawing his information from the fountain head, observing, that 'the specific name Tabacum is not, as was long supposed, a slight corruption of Tobago, or Tobasco, whence the drug is brought, but is, *as Humboldt has shown*, the Haytian word for the pipe."—*Outlines of Botany*, vol. ii.

cum,) hauriant, ut tanquam exanimes in terram concidant ibique maximam diei partem aut noctis velut stupefactis sensibus et capti mente jaceant.' And Lery relates, that (in Brazil, in S. Lat. 22½°,) the natives used merely a large tobacco leaf to involve several smaller ones: and thus as far as we can discover, was formed, for the first time, that pest of modern society—the cigar."* 10.

Adieu, then, to all hope of originality in Europe. We have been anticipated on every side, and, humiliating as is the reflection, we are reduced, after all, to smoke or snuff, to chew, and spit as our master, the Red Indian, did some three hundred years ago.

*Importation of Tobacco into Europe.*—We are naturally curious about this great event. The change of a dynasty sinks into insignificance beside it. There is no doubt that to Spain or Portugal belongs the honour of having first received the precious consignment. The first authentic account of its arrival, places that about the year 1558, when it was brought by a physician named Francisco Hernandes, who had been travelling for several years in Mexico. In the following year, Jean Nicot, Lord of Villemaine, on being sent as French Ambassador to Portugal, was presented with some of the herb, which by experiment he found was salutary in several diseases,—indeed, it was to an accidental application of the fresh juice to a tetter on the cheek, that his attention is said to have been first directed to the plant. After treating with success several of the inhabitants of Lisbon, who seem previously not to have been well aware of its properties, Nicot, in the year 1561, sent presents of some of it to Catherine de Medici—to the Grand Prior, and to several people of rank in France.

It is on this account that the herb has been called *Nicotiane—Herbe a l' Ambassadeur—Herbe à la Reine—Herbe du Grand Prieur.* Dr. Cleland entertains a very low opinion of French accuracy and French industry. In a note he observes that—"the French writers seem to be equally ignorant of the history of their own country and of ours. For example: Merat, (Dict. des Sc. Med. l. c.) after mentioning the importation by Nicot, in 1661, continues, 'mais il paroit que François Drak fameux amiral Anglais qui conquit la Virginie,'—had made it known in England before the period at which it was brought into France,—a circumstance rather astonishing, when it is recollected that at that very day Drake was a sailor in a coasting vessel betwixt England and Ireland, and that he had then only attained his fifteenth year."

In Europe, so ignorant were men that they actually looked on immortal tobacco as only fit for a medicine! Snuffing came first into vogue on account of having been employed as a sternutatory in some ophthalmic disorder, with which Charles IX. was affected; but it does not appear to have advanced with very rapid strides, until two reigns afterwards, viz. under that of Louis XIII, when it attained an almost universal employment. In France, smoking was much longer of being introduced, and at first it was through long straws, terminated by a little chafing dish of silver. Singular to re-

* "The Indian females, who suffered from attacks of Hysteria, were in the habit of carrying about with them little horns made of palm or cane, suspended from the neck, through which they inhaled, when attacked, the fumes of tobacco. *Ephem. Med. Phys. Acad. Germ.* D. ii. A. ii. Obs. 161."

late, the French have always regarded chewing as a barbarous practice. What an amount of rational enjoyment that lively people must have lost.

Perhaps nothing can, or ought to interest us more, than the epoch of tobacco reaching England. It is generally believed that this was in the year 1586, when it was brought to this country by some voyagers who had returned from a residence in Virginia; or, as that country was originally designated, Wingandacoa. Although, however, the majority of writers agree about the date, still there has arisen much disputation regarding the individual to whom should be assigned the title of introducer; and by different writers we find the claims of Raleigh, Lane, Drake, Greenville, and Harriot, respectively supported. Now it seems that Sir Walter Raleigh's second "adventure" to Virginia set sail on the 9th of April, 1585, and along with it Sir Walter sent Mr. Thomas Harriot, "a learned author and famous mathematician," as historian of the countries which the mariners might visit. This duty, accordingly, when he returned to England, in the following year, he set about performing; and in a short time afterwards published a work on the customs of the inhabitants of Virginia, and on the advantages which might be derived by cultivation of the country. In this work we observe the first mention of clay pipes for inhaling the smoke of tobacco; and in a plate which is subjoined in another part of the collection of De Bry, their form very nearly resembles that of those at present in use. Harriot enlarges much on the virtues of this herb; concluding his eulogium with the remark, that, those who employ it are not only freed from all kinds of obstructions in the system, but are, in addition, cured of those which they might chance to have, even though the complaint be of long standing." Master Harriot would seem, however, to have taken a spite towards tobacco subsequently, for in his Journal, quoted by Knickerbocker, he says of the Susquehanocks—"Their tobacco pipes were three quarters of a yard long, carved at the great end with a bird, beare, or other device, sufficient to beat out the brains of a horse! (*and how many asses brains are beaten out, or rather men's brains smoked out, and asses brains haled in by our lesser pipes at home!*") Very unmannerly language Master Harriot.

But Dr. Cleland feels disposed to place the precise period of the introduction into England betwixt the year 1563 and 1568, principally from the fact, that Sir John Hawkins, afterwards Treasurer of the Navy, returned during that period from several voyages during the course of which he had landed both on the coasts of Africa and Hispaniola, and whose scrutinizing observation it is very astonishing such a novelty should have escaped. And in confirmation of, or rather, as corroborated by this circumstance, is the express statement which the Water Poet, in one of his numerous productions makes; that to Sir John belonged the honour of making Britons acquainted with tobacco.

Whenever introduced, Sir Walter's influence, his precept and example too, helped smoking on a pace. And perhaps the Virgin Queen took her social pipe with the great ladies of her court, or, at all events with some of the numerous "fancy men," with whom that chaste dame disported. Whether Elizabeth smoked or chewed (and her black teeth were the admiration of some curious foreigners), in about ten years after its importation into England smoking was all the rage. Ben Jonson and Bishop Hall declaim against it handsomely.

Ben Jonson is particularly hard upon the smokers, though at his club, which met in the Apollo room at the Old Devil Tavern, there is reason to believe that the choice spirits did not disdain to "blow a cloud." And if surly Ben did wage war upon the weed, it had its patrons notwithstanding. The smart gallants—the "young England" of that day—

Quaffed a whole tunnell of tobacco smoke, the doctors patronised it, as we see from Nash's "Lenten Stuffe," published in 1599:—"Physicians deafen our ears with the honorificabilitudinitatis of their heavenly panacea—their sovereign guiacum—their clysters—their treacles—their mithridates, compacted of forty several poisons—their bitter rhubarb and torturing stibium. Amongst our English harmonious Calinos, one writes passing enamorately of the nature of white meats; a seventh *sets a tobacco pipe instead of a trumpet to his mouth, and of that divine drug proclaimeth miracles,*" and academies were even formed for teaching the taking of tobacco. In the play of Every Man Out of his Humour, a placard is represented on the wall of St. Paul's aisle, having the following contents:—"*If this city, or the suburbs of the same, do afford any young gentleman of the first, second, or third head, more or less, whose friends are but lately deceased, and whose lands are but new come into his hand, that, to be as exactly qualified as the best of our ordinary gallants are, is affected to entertain the most gentlemanlike use of tobacco; as first, to give it the most exquisite perfume, then to know all the delicate sweet forms for the assumption of it, as also the rare corollary and practice of the Cuban ebolition, Euripus and Whiffe, which he shall receive or take in here at London, and evaporate at Uxbridge, or farther, if it please him. If there be any such generous spirit, that is truly enamoured of these good faculties, may it please him but by a note of his hand, to specify the place or ordinary where he uses to eat and lie, and most sweet attendance with tobacco and pipes of the best sort, shall be ministered.* Stet, quæso, candide Lector." While Cavalier Shift declares, "It pleases the world, as I am her excellent tobacconist, to give me the style of Signior Whiffe. I do more than *profess*, sir, and if you please to be a *practitioner*, I will undertake in one fortnight to bring you that you shall take it plausibly in any ordinary, theatre, or the tilt-yard."

In those golden days there was none of that affectation of horror at tobacco, which our modern misses think so pretty. Then a man wooed and won with it, and his courtship was then literally, as it is now substantially, smoke.

Thus Fastidious Brisk, when paying his addresses to Saviolina, puffs out "Tobacco's smokie mists," in the interval of his high wrought speeches; and Sogliardo, about to court a lady, remarks to Macilente, "Faith an' you but say the word, I'll begin to her in tobacco." When tobacco was in such esteem

In prince's court and lady's bower, we need not be surprised that a monopoly was granted for the manufacture of tobacco pipes.

But hard times were at hand. Elizabeth was succeeded by the pedantic James who was no lover of smoking, and exorcised it as heartily as he did witchcraft. The kingly clerk blew his *counter blast to tobacco*, and blew it hard. Rip Van Winkle never smoked more lustily than puffed the anti-smoking monarch, who enumerates thus the ills that spring from the "rich

Indian vapour":—its serving as an incentive to drinking and lust,—its rendering the people so effeminate as to be unable to defend the kingdom,—its expensive character, ruining families,—its being disagreeable to associates, so that 'people of judgment' are obliged to begin it in self-defence,—and, lastly, its being inhaled into the brain, and so covering the surface with a black crust of soot." This latter assertion is rather startling, it is true, yet something may be said for it when we see the sable stream distilling from a veteran nose, and when we learn that James gives an account of the brains of individuals who had, during life, been excessive smokers, having been found, after death, covered with a black matter, which was very naturally presumed to be the remains of the volatilised tobacco. Much as this observation redounds both to the royal sense and veracity, James has not the exclusive credit of it, for Hoffman says, that the heads of some executed criminals (who had been great snuffers,) being dissected, the *patera* of the brain was black with snuff; and he was informed that the heads of the English soldiers who were killed in the Bohemian war, all who snuffed, had their brain in that condition. Unhappy men of snuff, when king and doctor manufactured such enormous lies against you.

James visited tobacco with something almost as heavy as the *counterblaste,* a tax of six shillings and eight pence. This was the unkindest cut of all.

At the primary introduction of tobacco into Great Britain, and for some time afterwards, no commercialist seems to have exclusively devoted himself to the vending of this herb; supplies of it being in general obtained from the grocers.

" In process of time, the sale of it devolved upon the apothecary; and, at a still later period, it became an article of profit to the tavern keeper. In the year 1610, however, when the *Alchemist* was published, we find that *honest Abel's* shop was exclusively a divan where, as *Face* declares, he keeps his tobacco—

> ' In fine lily pots that, open'd,
> Smell like conserve of roses, or French beans,—
> He has his maple block, his silver tongs,
> Winchester pipes, and fires of juniper.'

But, notwithstanding the increase in the amount necessary to procure tobacco, the demand for it, although for a short time somewhat diminished, appears soon to have returned to its former extent, or even to have surpassed it. For example, in the '*Devil is an Ass,*' 'carmen' are said to have 'got into the yellow starch, and *chimney-sweepers* to their *tobacco* and strong waters.' "

There is nothing new under the sun, and if we see now-a-days a dustman take his cheroot, he does little more than generations of dustmen did before he was born. It seems that the heads of three of the four great divisions of religionists have anathematised the weed. The Pope and the Puritan have damned it alike, and with almost equal cordiality, and in their violence they have supported the clerical character. Zachary Boyd excludes the smoker from Heaven, and another preacher says:—" the people cannot wait until the smoke of the infernal regions surrounds them, but encompass themselves with smoke of their own accord, and drink a poison which God made black that it might bear the devil's colour." And what do our readers think this infernal mixture may be?—Coffee! Hear this, ye temperance-men—rejoice, publicans—tobacco and coffee are damned together.

Tant de fiel entre t'il dans l'ame des devots?

But there are kindlier feelings afloat towards the maligned herb. The author of a "Paper of Tobacco," takes a fairer view of its deserts, and does something like justice to its powers. He informs us that the greater facility with which the Scotch acquire the French language than the inhabitants of England, is "undoubtedly to be ascribed" to the nasal twang which, in his opinion, is imparted by the use of snuff; a statement which completely puts in the shade every educational scheme or mode from the days of Adam to those of Pestalozzi.

"In many cases of religious melancholy, where long prayers are ineffectual, great relief," it is remarked, "may often be expected from a *short pipe*. The value of tobacco at lyk-wakes is well known in every part of the United Kingdom. It *blunts the edge of grief*, and *by inducing kindly feelings*, causes the neighbours and friends of the deceased *to forget his faults and to enlarge upon his good qualities*." This is handsome. And indeed it is only fair to the church to confess that if some of its members have been virulent, the great majority have been rather of Parson Adams' way of thinking, and have looked upon their pipes as comfortable things. This is rightly taken notice of in a reply to a supposed appeal by the chaplains of the kingdom to Parliament, wherein

"Besides store of dishes—
One part of their wishes—
To fortify maw sacerdotal,
Eleemosinary Funk,
And leave to be drunk,
*They* humbly desire you to vote all,"

it is declared, that, "as to the chaplains, we are willing to allow them plenty of meat, drink, and *tobacco, the most zealous* part of their supplication."

Our author entertains the interesting question whether a *quid* or a *pinch* was first patronised in England. The Duke of Albemarle (Monk) certainly took his quid. Pepys notices this. "Sir William Coventry," observes this quaint chronicler of *ladies' patches* and *exceeding base dinners*," spoke slighting of the Duke of Albemarle, saying, when De Ruyter came to give him a broadside, says *he, (chewing of tobacco the while,)* will this fellow come and give me two broadsides and then he will run." And the custom was so general among the English as to induce a German of the name of Junker, to entitle a book which he wrote, "*Dissertatio de masticatione Tabaci (of schawing Tobacco) in Anglia usitatâ.*"

But, in good Queen Anne's reign, snuff took the lead. Even the ladies adored it, the box being as necessary an article of the fashionable toilet as the oil of talc, or the ceruse pot; and the present of a *tabatiere* being universally looked upon as a tender of affection.*

We need not pursue the introduction of the precious herb into other countries, nor dilate on the just sense of its merits which the German and the

* "LORD FOPPINGTON.—Lady Betty was just upon the wing; but I caught her by *the snuff-box,* and she pretends to stay to see if I will give it her or no.

LORD MORELOVE.—Death! 'tis that I gave her, and the *only present she would ever receive from me.*"—*The Careless Husband,*—*Act* iii. *Scene* ii., 1700.

Hollander evince. Whoever has had the good fortune to ride inside a German or a Belgic diligence, must recollect the fragrant scent from yesterday's tobacco and to-day's; and he must deprecate the squeamishness which in this country interferes with men smoking at all times, in all places, and in every company.

Nor need we pursue the ungrateful task of chronicling the unsuccessful attempts that have at different times and in different countries been made to put it down. Men without souls for a cheerful pipe have preached crusades against it, and by fine, and imprisonment, and even death, have waged a cruel war with smoke. Smoke, however, has vanquished them, and rises triumphant while its opponents are laid low. In vain has the lawyer, in vain has the divine attempted its overthrow in our happy island—by a signal retribution ignominy has covered the memory of the kingly blower of the *counterblaste*—and we stand pre-eminent among the nations as a snuffing, a chewing, and a smoking race. Happy England, "great, glorious, and free," in whose metropolis the learned Pigot, he of the Directory, informs us there are 1,200 venders of tobacco, and only 2,200 bakers. Verily if John Bull deems bread the staff of life, he looks upon tobacco as the joy of it.

*Analysis of Tobacco.*—This must necessarily attract a high degree of interest. We need not be surprised at the contradictory statements of various chemists, for every one who knows anything of the history of organic chemistry is aware of the discrepancies that obtain in it. We therefore take the last analysis, as, of course, the least contradicted—the analysis of MM. Henri and Boutron Charlard. They, in 1836, by distilling *Tabac à fumee* twice off caustic soda, and by placing the product, of a syrupy consistence, in a vacuum, obtained a substance bearing a considerable resemblance to chlorate of potass, and which they designated *Nicotine.* This alkaloid has no odour when cold, but when heat is applied it evaporates completely away, being transformed into a white irritating smoke, having a smell like that of tobacco, and even when much diluted, the taste is very harsh, acrid, and caustic. It is soluble in ether, alcohol, turpentine, water, and weak acids. It forms salts with acids, which are decomposed partially, even at a moderate temperature. Caustic soda causes nicotine to give off ammonia, and by the *strong* mineral acids it is completely destroyed. As it contains more than twice the quantity of azote which quinine does, it must be considered a very powerful base.

As tannin forms a very insoluble salt with nicotine, astringent substances are pointed out as the proper agents to be had recourse to, when a case of poisoning by means of this occurs.

This substance is exceedingly poisonous, being only inferior in point of rapidity to arsenietted hydrogen. On a drop of the solution being introduced into the beak of a strong pigeon, *la foudroye instantanément,* and small birds were killed in a moment merely by being brought near a tube impregnated with it. Henri having through inadvertence drawn into the mouth, for a second or two, an exceedingly weak solution of nicotine "experienced a violent stunning sensation, (etourdissiment) to which succeeded a sense of weight, pain, and oppression in the head, continuing for several hours." It is this substance which is the real cause of the poisonous effects so often mentioned by old writers, as caused by the *oil* of tobacco.

*Action of Tobacco.*—It was soon found to be, in the main, a *sedative.* Lord Bacon considered the powerful consequences to depend upon a *condensation* of the spirits, without however alluding to any previous abnormal *expansion.* But perhaps we ought mostly to admire Willis's notion of the spirits being driven back by tobacco from the centre to the cortex of the brain, like a defeated army in consternation, whereby the soul being more contracted, retiring into itself lies down in rest, just almost in the same manner as throwing water upon a fire that breaks vehemently out *immediately* beats down the aspiring flame.

Be this as it may, the following are the effects of tobacco. On the reception of a moderate dose, the secretions are for the most part increased, especially the mucous surfaces of the intestinal and urinary organs, and even the skin is to a small extent interested. These phenomena are gradually increased in intensity on a larger quantity of the medicine being administered, until diarrhœa and vomiting, accompanied with nausea, relaxation of the muscular fibre, tottering of the limbs, and very great depression of strength supervene. The pulse during this stage is very small, weak, and even intermitting; the face is blanched and contracted, frequently bathed in cold perspiration, while at the same time, the mind is in a very depressed condition, and frequently complete syncope winds up the train of symptoms. In the third stage of symptoms caused by tobacco, viz. that of poisoning, the breathing becomes very difficult, the limbs are alternately convulsed and paralysed, the muscles of the neck frequently affected with tetanic spasm; the pulse becomes weak, slow, and fluttering; the pupil is contracted and insensible; a universal rigour prevails in every part; and the patient is in general cut off during the existence of a paroxysm.

Short, however, of this, ill-natured persons say that tobacco spoils digestion. Every body knows that a quid is a cheap meal, and some who smoke are not much the hungrier for it. Old smokers, we have observed, are no great eaters, labour under dyspepsia, and are apt to have scaly eruptions on the skin. The effect on the appetite has long been known, and even embodied in verse:—

"All dainty meats I do defy,
 Which feed men fat as swine,
He is a frugal man indeed,
 That on a leaf can dine.
He needs no napkin for his hand,
 His finger ends to wipe,
That keeps his kitchen in a box,
 And roast meat in a pipe."

*Sanatory Effects of Tobacco.*—Our forefathers set a great value on them, and with reason. For, "in the first place," says Chrysostom Magnenus, "the manner in which the flowers adhere to the head of the plant indicates the *Infundibulum Cerebri* and *Pituitary Gland.* In the next place, the three membranes of which its leaves are composed announce their value to the stomach, which has three membranes."

Dr. Cleland observes that as the maladies for the alleviation of which it has been recommended and administered embrace those contained in almost every genus, order, and class of Cullen, we cannot do better than subjoin the nosology. After this it would be superfluous to go on. From toothache to tetanus, and from Chrysostom Magnenus to Dr. O'Beirne is a jump wide enough in all conscience, at all events enough for us. And so we conclude, wishing all good to the "weed," plenty of it to our readers, Havannahs of the finest quality to Dr. Cleland, and Theses as entertaining and as instructive withal from every candidate for the Doctor's degree in the University of Glasgow.

